# Association of brain immune genes with social behavior of inbred mouse strains

**DOI:** 10.1186/s12974-015-0297-5

**Published:** 2015-04-18

**Authors:** Li Ma, Sami Piirainen, Natalia Kulesskaya, Heikki Rauvala, Li Tian

**Affiliations:** Neuroscience Center, University of Helsinki, Viikinkaari 4, FIN-00014 Helsinki, Finland; Psychiatry Research Center, Beijing Huilongguan Hospital, Zhengfu Road, 100096 Beijing, Changping China

**Keywords:** Social deficit, Inbred mouse strains, Immune genes, Behaviors, Brain morphology

## Abstract

**Background:**

Social deficit is one of the core symptoms of neuropsychiatric diseases, in which immune genes play an important role. Although a few immune genes have been shown to regulate social and emotional behaviors, how immune gene network(s) may jointly regulate sociability has not been investigated so far.

**Methods:**

To decipher the potential immune-mediated mechanisms underlying social behavior, we first studied the brain microarray data of eight inbred mouse strains with known variations in social behavior and retrieved the differentially expressed immune genes. We then made a protein-protein interaction analysis of them to find the major networks and explored the potential association of these genes with the behavior and brain morphology in the mouse phenome database. To validate the expression and function of the candidate immune genes, we selected the C57BL/6 J and DBA/2 J strains among the eight inbred strains, compared their social behaviors in resident-intruder and 3-chambered social tests and the mRNA levels of these genes, and analyzed the correlations of these genes with the social behaviors.

**Results:**

A group of immune genes were differentially expressed in the brains of these mouse strains. The representative C57BL/6 J and DBA/2 J strains displayed significant differences in social behaviors, DBA/2 J mice being less active in social dominance and social interaction than C57BL/6 J mice. The mRNA levels of *H2-d1* in the prefrontal cortex, hippocampus, and hypothalamus and *C1qb* in the hippocampus of the DBA/2 J strain were significantly down-regulated as compared to those in the C57BL/6 J strain. In contrast, *Polr3b* in the hippocampus and *Tnfsf13b* in the prefrontal cortex of the DBA/2 J strain were up-regulated. Furthermore, *C1qb*, *Cx3cl1*, *H2-d1*, *H2-k1*, *Polr3b*, and *Tnfsf13b* were predicted to be associated with various behavioral and brain morphological features across the eight inbred strains. Importantly, the *C1qb* mRNA level was confirmed to be significantly correlated with the sociability in DBA/2 J but not in C57BL/6 J mice.

**Conclusions:**

Our study provided evidence on the association of immune gene network(s) with the brain development and behavior in animals and revealed neurobiological functions of novel brain immune genes that may contribute to social deficiency in animal models of neuropsychiatric disorders.

## Background

Increasing evidence highlights the involvement of immune dysfunction in the complex mental diseases, such as schizophrenia and autism spectrum disorder, in which genetic predisposition(s) requires additional environmental impact(s) to determine the progression of pathophysiology [1,2]. Maternal immune activation has been shown to induce neuroinflammation and schizophrenic/autistic phenotypes in both humans and other species [3-8]. Accumulating genetic evidence has recently also provided support on the association of immune genes with psychiatric diseases [1,9]. Among the major disease manifestations, social deficit is a core symptom that negatively influences the lives of psychiatric patients. In fact, social behavior has been intimately associated with infections and inflammatory diseases in human evolution [10-12].

In animal research, a handful of studies have revealed the association of immune activation and immune genes with social and emotional behaviors [13-16]. For instance, triggering immune defenses in pregnant monkeys can lead to social problems in their offspring [17]. Recently, microglia as a major source of immune genes in the brain has been extensively reported to regulate the brain functional connectivity and behavior [18-21]. Moreover, immune-related genes, such as the major histocompatibility complex (MHC) molecules, complements and their receptors, are known to be expressed in the brain and regulate brain structural and functional plasticity, either directly or indirectly by controlling microglial or immune activation [22-24]. Therefore, the potential association of social behavior with immune genes in the brain becomes an outstanding question. However, although a few immune genes have been discovered, it is predictable that a complex social behavior is associated with not only a singular, but rather a network of different genes that may have a relatively weak effect size individually. In this respect, addressing how various immune genes may jointly regulate sociability is of upmost relevance and importance but has not been fully investigated so far.

Due to ethical and empirical limitations in clinical studies, rodent models have proven to be highly useful in characterizing the mechanisms of psychiatric diseases. Multiple genetically different inbred mouse strains exist and many exhibit clear and reliable differences in learning and memory [25], sensorimotor gating [26], and anxiety [25,27,28], resembling the neuropsychiatric endophenotypes [28-30]. Such strain differences offer clues to decipher genetic and gene × environmental influences on behaviors. In this regard, we have recently used such strategy to characterize the association of microglial activation with anxiety [28]. Deficit in sociability may also represent a key feature among some of these inbred strains. However, approaches used to study social behaviors and results on inbred mouse strains are still very limited. A previous study suggested less significant sociability and social novelty preference of the male DBA/2 J strain than the C57BL/6 J strain [31]. Similarly, we recently found that female DBA/2 J mice were significantly less active in social interaction, as compared to C57BL/6 J female mice, but we had not explored male mice in that study [32]. It is therefore necessary to more fully characterize the differences in social behaviors of inbred strains and to explore further the usefulness of this strategy to discover the underlying mechanisms.

To more elaborately address the immune-mediated mechanisms in social deficiency, we took an unbiased evidence-driven strategy by analyzing the publicly available genotypic and phenotypic data of several male inbred mouse strains with bioinformatics approaches. We first made the gene expression profiling by using online brain DNA microarray data and selected candidate genes for functional clustering analysis. To predict the association of the interested genes with the brain development and behaviors, we aligned their expression data with the online behavioral data. Lastly, we examined the C57BL/6 J and DBA/2 J mouse strains that displayed significant differences in social behaviors to validate the expression of the major candidate genes and their association with social behaviors.

## Materials and methods

### Animals

Seven-week-old male mice from two inbred strains, C57BL/6 J (*n* = 8) and DBA/2 J (*n* = 8), were purchased from a single supplier, Harlan Laboratories (The Netherlands), to avoid a possible source of variation. The mice were single-housed in standard cages with aspen chips bedding and nesting materials, food, and water available *ad libitum* and under a 12-h light/dark cycle (lights on 6.00 to 18.00). Animals were acclimated to the environment for 3 weeks before experiments. The research was performed with permission by the National Animal Experiment Board of Finland.

### Resident-intruder test

For assessment of reciprocal social behavior, tested animals (residents) were in their home cages. An unfamiliar intruder mouse of the same strain, gender, and age (Harlan Laboratories) was placed into the resident’s cage, and mice interaction was recorded by a video camera for 5 min. Time spent by the resident in social behavior (sniffing, chasing, following, and heterogrooming) was evaluated.

### Three-chambered sociability test

The inbred mice were individually tested in a three-chambered apparatus as described previously [33]. The test apparatus consisted of three rectangular chambers (18 × 35 × 18 cm) divided by Plexiglas walls with openings (6 × 5 cm) allowing the animal to move between chambers. Both side chambers contained an empty transparent Plexiglas holder (8 cm in diameter, 10 cm high, with small holes allowing snout contact but not biting or fighting between the animals). A test mouse was first released in the central chamber and was allowed to habituate to the apparatus for 10 min. An unfamiliar gender- and age-matched stranger mouse of the FVB strain (Harlan Laboratories) was then placed in one of the holders. Location of the stranger mouse in either of the two holders (the social chamber) varied systematically between trials. The test mouse was allowed to explore the whole apparatus for 10 min. Time spent in sniffing of each holder was recorded by a video camera.

### Inbred mice brain microarray data processing and differential expression analysis

Whole brain microarray datasets of 10 ~ 12-week old male mice of eight inbred strains (129S1/SvImJ: *n* = 5, A/J: *n* = 4, BALB/cByJ: *n* = 5, C3H/HeJ: *n* = 5, C57BL/6 J: *n* = 5, DBA/2 J: *n* = 5, FVB/NJ: *n* = 5, and SJL/J: *n* = 4), which were produced in an Affymetrix 3’ 430_2.0 platform [34], were selected from the PhenoGen Informatics Database (University of Colorado and Denver Health Sciences Center, Denver, CO, USA) after quality control of their integrities. Arrays were grouped according to the strains and normalized by the RMA method. Differential expression was done by using the analytical tool in PhenoGen Informatics. Probes in the dataset were first filtered through the MSA5 absolute call filter, and those present in at least 75% of the samples were kept. One-way analysis of variance (ANOVA) was used for statistical evaluation. *P* values were further adjusted with the Benjamini and Hochberg method for multiple test *post hoc* correction with FDR *=* 1E − 7. A list of differentially expressed genes and their statistical data were finally downloaded from Phenogen Informatics for further bioinformatics analyses.

### Functional annotation clustering of differentially expressed brain genes and protein-protein interaction network analysis

Differentially expressed genes were imported into the Database for Annotation, Visualization and Integrated Discovery (DAVID) Bioinformatics Resources [35] and analyzed by the functional annotation tool therein to get the most significant gene clusters according to their gene ontology terms (GO_biological process, cellular component, and molecular function). Clustering of annotated genes was done with the medium classification stringency and under the default statistical settings. Twenty-three top innate immune genes were further analyzed for their direct and indirect protein-protein interaction networks by the STRING v9.1 [36] and for their phenotypic correlations in the Mouse Phenome Database (MPD) [37].

### Online correlational analysis of immune genes with mice behaviors and brain morphology

Mean expression values of the brain immune genes across the eight inbred mouse strains were fed into MPD’s correlational analysis tool (Pearson’s), to be aligned with the data on mouse psychiatry-related behaviors, including anxiety-related, exploratory, fear conditioning, learning and social behaviors, and on brain morphologies. Pearson’s co-efficients were set at |*r|* ≥ 0.7, *P* < 0.05. Behavioral and brain morphological data on the eight inbred strains were then retrieved from the MPD with the corresponding registration numbers (MPD #118 for anxiety, #108 for brain morphology, #94 for exploratory behavior, #468 for fear conditioning) [38-43].

### Total RNA isolation and real-time qPCR

The mice were deeply anesthetized with pentobarbital (Orion Pharma, Helsinki, Finland) for about 5 min, and the prefrontal cortex (PFC), hypothalamus, and hippocampus were dissected after intra-cardiac perfusion, immediately frozen in liquid nitrogen, and kept at −80°C. Total RNAs from the tissues were extracted by using GeneJET RNA Purification Kit (Thermo Scientific (Inc.), Helsinki, Finland) and reversely transcribed (1 μg) with RevertAid First Strand cDNA Synthesis Kit (Thermo Scientific). RT-qPCR was performed by using corresponding primers and Maxima SYBR Green Master Mixes (Thermo Scientific) on a CFX384 Real-Time PCR Detection System (Bio-Rad, Helsinki, Finland) according to the manufacturer’s instructions. The forward and reverse primers used for target genes were listed below. Relative fold change was calculated by first normalization with the reference gene *Gapdh* and then against the level in C57BL/6 J and presented as 2-ΔΔCT. No strain difference in *Gapdh* and another reference gene *Actb* was observed (data not shown).

### Gene Primers

*Tnfsf13b* For: 5′-GAC TGT CTG CAG CTG ATT GC-3′

Rev: 5′-CCT CCA AGG CAT TTC CTC TT-3′

*C1qb* For: 5′-TCT GGG AAT CCA CTG CTG TC-3′

Rev: 5′-AGA CCT CAC CCC ACT GTG TC-3′

*H2-d1* For: 5′-GATGCAGAGCATTACAGGGC-3′

Rev: 5′-GCCAGGTCAGGGCAATGTC-3′

*Polr3b* For: 5′-AGACAAATTCAGCAGTCGCC-3′

Rev: 5′-GGGTTCATTATGATGTCGGG-3′

*Gapdh* For: 5′-TGT TCC TAC CCC CAA TGT GT-3′

Rev: 5′-TGT GAG GGA GAT GCT CAG TG-3′

### Statistical analysis

Normality was evaluated by the D’Agostino and Pearson omnibus test. Student’s *t* test was used for analysis of the resident-intruder and three-chambered test data. qPCR data for immune genes were analyzed by two-way analysis of variance (ANOVA) with Bonferroni’s *post hoc* test. Correlations of the candidate gene mRNA levels with the percentages of social time in the resident-intruder and three-chambered tests were analyzed by Pearson’s method. Results are presented as mean ± SEM. *P* < 0.05 was considered to be significant.

## Results

### Various immune genes are differentially expressed in the brains of inbred mouse strains

To unbiasedly find out whether immune genes are expressed in a strain-specific manner, we compared the brain gene expression data from Affymetrix microarrays. We chose male mice of the eight inbred strains in our study, 129S1/SvImJ, A/J, BALB/cByJ, C3H/HeJ, C57BL/6 J, DBA/2 J, FVB/NJ, and SJL/J, which are the most commonly used strains in genetic, pharmacological, and behavioral studies on neuropsychiatric disorders. All these strains differ in their emotional, exploratory, and cognitive functions and belong to the “group A” priority strains in the Mouse Phenome Project. Altogether, 2,983 differentially expressed (selected at FDR *=* 1E − 7) probesets (representing 2,584 mouse genes that DAVID identifies) among the eight strains were discovered. To better grasp the molecular functions, cellular localizations, and biological processes that they are involved in, we retrieved a list of these genes and imported it into DAVID for functional annotation clustering based on their GO terms. The top three most significant clusters of genes belong to cytoskeletal, synaptic junction, and vesicular molecules (data not shown).

**Figure 1:**
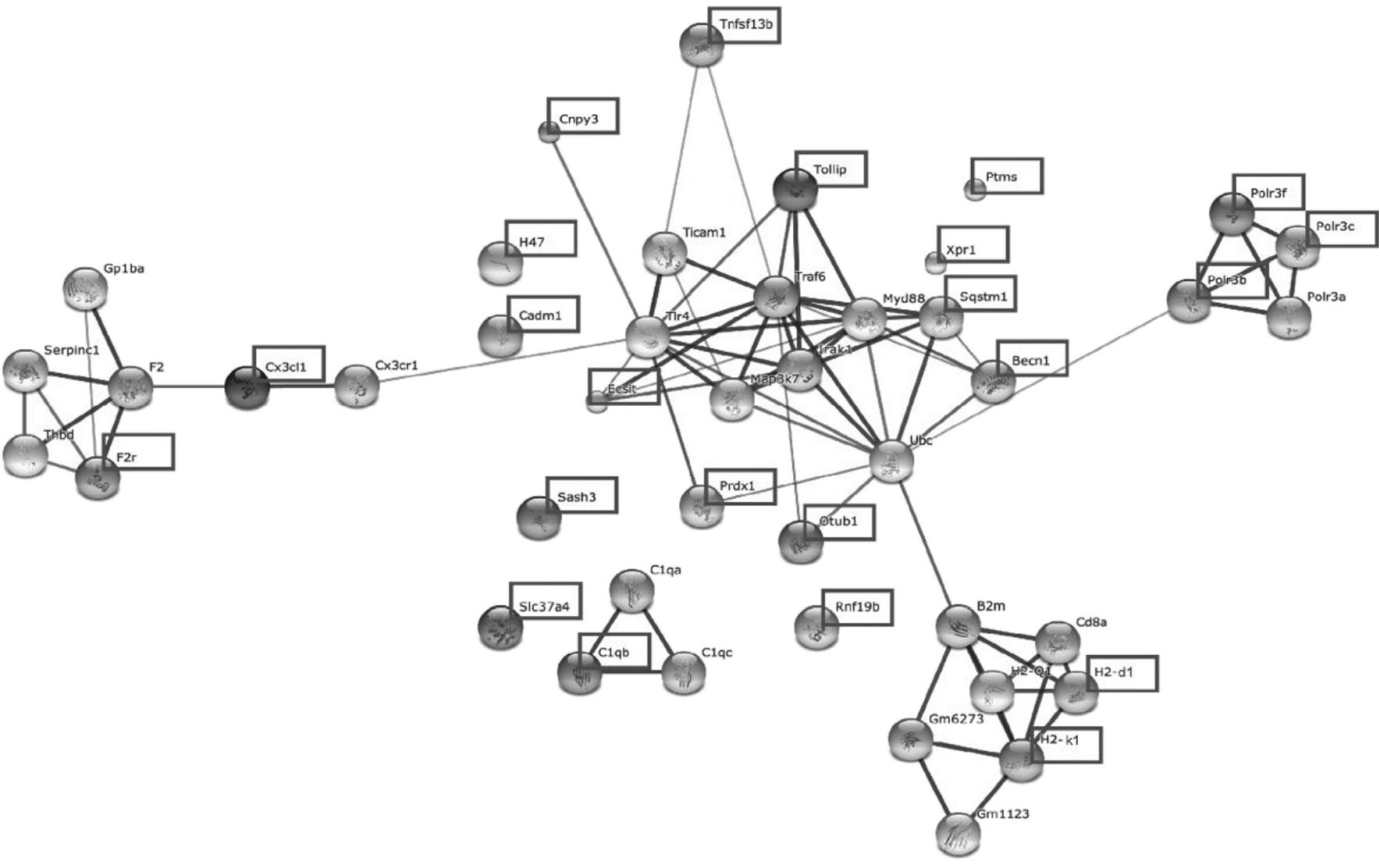
Protein-protein interaction network analysis of differentially expressed innate immune genes among inbred mouse strains with STRINGv9.1. Five major clusters were identified from the 23 immune genes (marked by squares) discovered through microarray analysis of brain genes in 8 inbred mouse strains. Confidence of an interaction is represented by the thickness of the line.

Among the 2,584 genes that DAVID identified, we found altogether 83 immune genes, out of which 23 genes (with 35 probesets in microarrays, representing about 1% of the total differentially expressed gene population and marked in Figure 1 by squares) have innate immune functions. To better understand the functional relationships of these 23 immune genes, we further used the STRING v9.1 to identify protein-protein interaction networks among these genes. The analysis revealed five clusters, which include genes that belong to coagulation factors (*F2r*), MHC class I family (*H2-d1* and *H2-k1*), tumor necrosis factor family (*Tollip* and *Tnfsf13b*), antiviral molecules (*Polr3b*, *Polr3c* and *Polr3f*), and complement components and receptors (*C1qb* and *Cd47*). In addition, *Cx3cl1* has a strong association with *F2* (Figure 1).

### Prediction of immune-associated behavioral and brain morphological changes of inbred mouse strains

To further depict the functional meaning of these 23 immune genes and to find out the types of behaviors they may be involved in, we explored the MPD database, which contains a vast repository of various biological data on these strains. By using the correlational tool therein, as described in the Materials and methods section, we found a list of significantly correlated phenotypes for the genes *C1qb*, *Cx3cl1*, *H2-d1*, *H2-k1*, *Polr3b*, and *Tnfsf13b*. The microarray relative expression levels of these six immune genes are summarized in Table 1. Among them, we found that several genes have the highest expression levels in the C57BL/6 J strain.

**Table 1:** **Differential expression analysis of selected brain immune genes across eight inbred mouse strains**

**Gene Symbol**	**129S1/SvImJ (** ***n*** **= 5)**	**A/J (** ***n*** **= 4)**	**BALB/cByJ (** ***n*** **= 5)**	**C3H/HeJ (** ***n*** **= 5)**	**C57BL/6 J (** ***n*** **= 5)**	**DBA/2 J (** ***n*** **= 9)**	**FVB/NJ (** ***n*** **= 5)**	**SJL/J (** ***n*** **= 4)**	**Adjusted** ***P*** **value**
*C1qb*	8.41	8.64	*7.91*	9.00	**9.71**	8.51	8.77	8.43	1.56E − 09
*Cx3cl1*	*9.95*	10.91	9.96	11.12	**11.33**	10.96	11.00	11.11	2.59E − 09
*H2-d1*	9.91	10.66	*9.53*	10.08	10.97	10.43	**11.76**	9.68	2.40E − 12
*H2-k1*	9.76	8.64	9.71	*8.06*	**10.65**	10.32	10.24	9.96	5.20E − 24
*Tnfsf13b*	6.53	6.17	**6.63**	5.94	*5.91*	6.40	6.09	6.09	5.42E − 08
*Polr3b*	7.41	*6.69*	7.38	**7.76**	7.49	7.56	7.65	7.39	4.35E − 09

Microarray data of the eight inbred male mice brains were retrieved and analyzed for differential expression in Phenogen Informatics. One-way-ANOVA was used as the statistical method. *P* values were further corrected by the Benjamini and Hochberg method for multiple test *post hoc* correction with FDR *=* 1E − 7. Mean expression levels are presented, and the highest and lowest levels for each gene are bold and italicized, respectively.

Since MPD currently provides very limited data on mouse social behavior *per se*, we focused on the data that are most related to social behavior, which are summarized in Table 2. Our analysis revealed that these genes may be significantly associated with various behaviors. For instance, the MHC gene *H2-k1* is positively associated with anxiety (*r* = 0.91, *P* = 0.03, Table 2), and *H2-d1* and *H2-k1* with exploratory activity (*r* = 0.81 ~ 0.91, *P* = 0.04 ~ 0.01, Table 2) of animals, based on the study by Fraser *et al*. (MPD #94) [41]. Furthermore, *C1qb*, *Cx3cl1*, and *Polr3b* are negatively associated with the time an animal spends near the walls of a conditioning chamber after contextual/cued fear conditioning (*r* = −0.91 ~ −0.97, *P* = 0.03 ~ 0.006, Table 2), based on the study by Park and colleagues (MPD #468) [42]. Such predictive data suggest that genes like *H2-d1*, *H2-k1*, *C1qb*, *Cx3cl1*, and *Polr3b* may be involved in the behavioral processes that are related to and inherently important for social activities of animals.

**Table 2:** **Meta-analysis of geno-phenotype correlation**

**Gene symbol**	**Behavior (MPD #)**			
	**Anxiety #118**	**Exploratory-locomotion #94**	**Fear conditioning #468**	**Brain morphology #108**
***C1qb***			−0.95 (0.01)	
***Cx3cl1***			−0.97 (0.006)	CC_0.89 (0.003)
***Polr3b***			−0.92 (0.0249)	
***H2-d1***		0.91	−0.95	
		(0.035)	(0.014)	
***H2-k1***	0.91	0.82		
	(0.032)	(0.013)		
***Tnfsf13b***				CC_ − 0.85 (0.008)

Phenotypic correlation of some immune genes with male mice behaviors and brain morphology was analyzed in mouse phenome database (MPD) with the Pearson’s method. MPD registry number for each study used in the analysis is listed. Pearson’s *r* values for the most significant correlations are summarized. *P* values are in parentheses in the table. Evaluation criteria used are the following: anxiety, entries into closed arm of elevated plus maze; exploratory-locomotor activity, entries into the center of elevated plus maze and distance traveled in open field; fear conditioning, time spent (%) near chamber walls in a contextual/cued fear-conditioning chamber; brain morphology, corpus callosum (CC) length.

Another interesting finding was that many of these immune genes are associated with the brain morphology (Table 2) (MPD #108) [39]. For example, *Cx3cl1* is positively associated with the length of the corpus callosum (CC) (*r =* 0.89, *P* = 0.003, Table 2). In contrast, the pro-inflammatory cytokine *Tnfsf13b* is negatively associated with the length of CC (*r =* −0.85, *P* = 0.008, Table 2). A detailed correlational analysis among the eight strains shows that the C57BL/6 J strain expresses the highest levels of *Cx3cl1* and the lowest level of *Tnfsf13b*, while containing the longest CC. In comparison, the 129S1/SvImJ strain expresses the lowest levels of *Cx3cl1* and the relatively higher level of *Tnfsf13b*, while containing the shortest CC (Table 1, data on CC length not shown).

### DBA/2 J mice exhibit social deficiency as compared to C57BL/6 J mice

**Figure 2:**
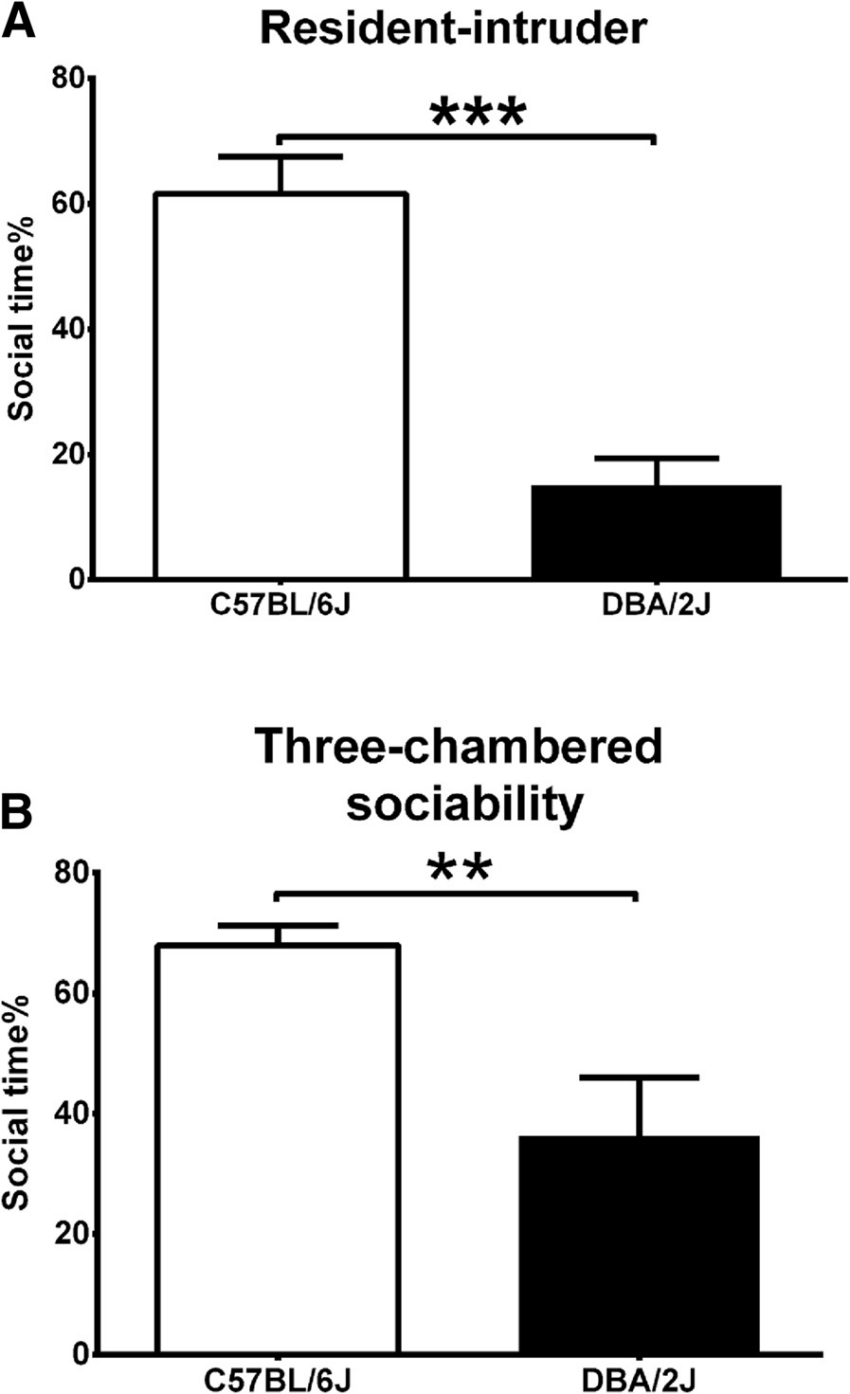
DBA/2 J mice show impaired social behavior. The percentages of social time spent by the DBA/2 J and C57BL/6 J mice in **(A)** resident-intruder test and **(B)** three-chambered test are shown (*n* = 8 per strain). **P < 0.01; ***P < 0.001. Data were analyzed by Student’s *t* test. Results are presented as mean ± SEM.

To validate the microarray data, we next selected two strains, DBA/2 J and C57BL/6 J, which have been most extensively studied in behavioral research and represent mice of low and high sociability, respectively [44]. Since resident-intruder and three-chambered social tests are the most commonly utilized ones to evaluate sociability in these studies, we took these two classical methods to first compare social behaviors between the male DBA/2 J and C57BL/6 J mice. In the resident-intruder test, the DBA/2 J mice spent less percentage of time in social interaction with intruders (*P* < 0.0001) (Figure 2A). In the three-chambered test, the DBA/2 J mice also exhibited less percentage of social time than the C57BL/6 J mice (*P* < 0.01) (Figure 2B). No difference in explorative activity between these two strains was observed in the three-chambered test (data not shown).

### Validation of several immune genes in the brains of C57BL/6 J and DBA/2 J mice

**Figure 3:**
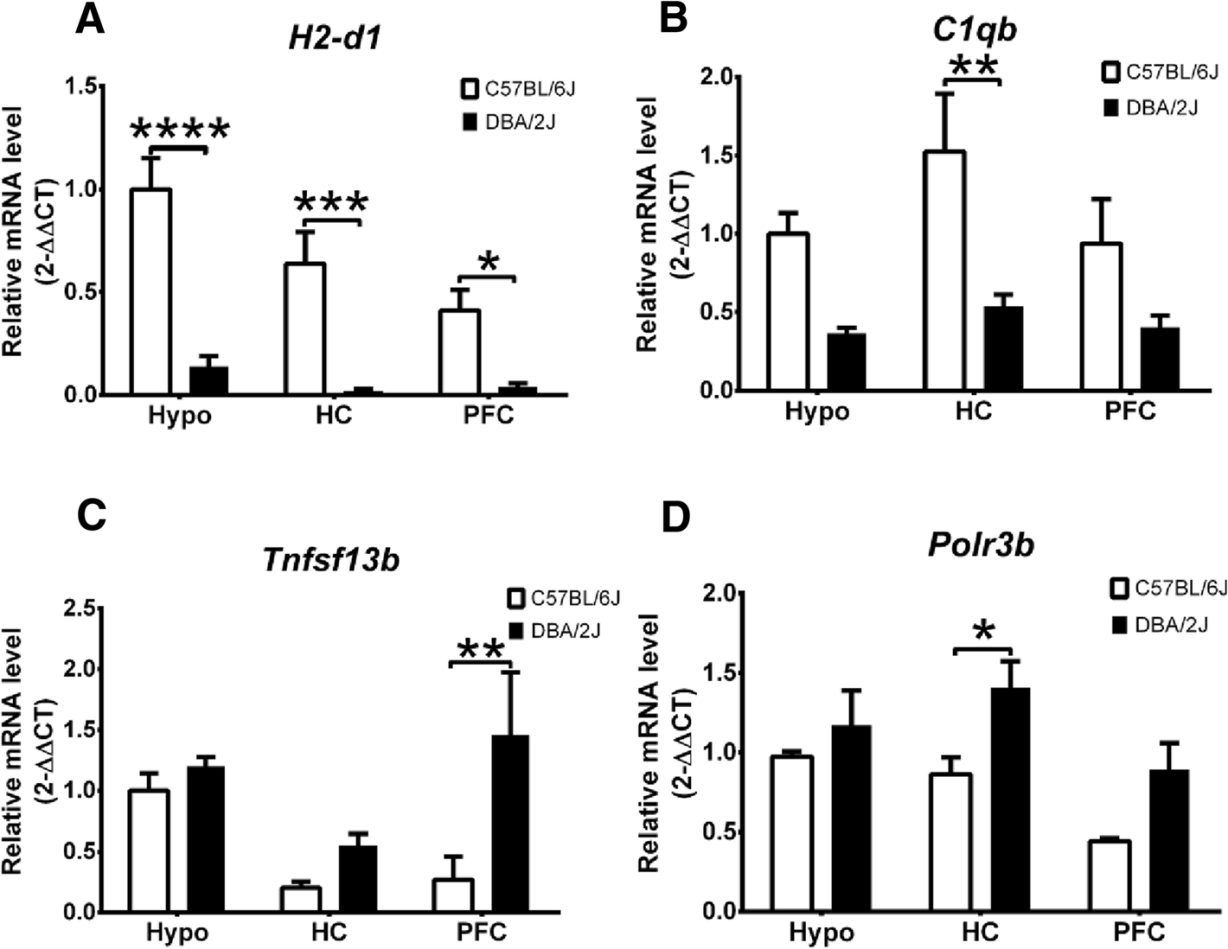
Differential expression of immune genes *H2-d1* **(A)**, *C1qb* **(B)**, *Tnfsf13b* **(C),** and *Polr3b* **(D)** in the prefrontal cortex (PFC), hippocampus (HC), and hypothalamus (Hypo) between DBA/2 J and C57BL/6 J strains by RT-qPCR. Relative expression level was first normalized against *Gapdh* and then set as fold change against the expression level in the hypothalamus of the C57BL/6 J mice for each gene (*n* = 8 per strain). Data were analyzed by two-way ANOVA with Bonferroni’s *post hoc* test. *P < 0.05; ** *P* < 0.01; ***P < 0.001; ****P < 0.0001. Results are presented as mean ± SEM.

We then validated the mRNA levels of the six immune genes *C1qb*, *Cx3cl1*, *H2-d1*, *H2-k1*, *Polr3b*, and *Tnfsf13b* by RT-qPCR in the PFC, hippocampus, and hypothalamus of the C57BL/6 J and DBA/2 J strains. No significant interactions between strain and region in the expression of these immune genes were observed. Of particular interest, the mRNA levels of *H2-d1* were significantly decreased in the PFC (*P* < 0.05), hippocampus (*P* < 0.001), and hypothalamus (*P* < 0.0001) of the DBA/2 J mice (Figure 3A). Similarly, the mRNA level of *C1qb* was decreased significantly in the hippocampus of the DBA/2 J mice (*P* < 0.01) (Figure 3B). In contrast, DBA/2 J expressed significantly higher mRNA level of *Tnfsf13b* in the PFC (*P* < 0.01) (Figure 3C) and that of *Polr3b* in the hippocampus (*P* < 0.05) (Figure 3D) than C57BL/6 J. However, no significant differences in the expression of *Cx3cl1* and *H2-k1* were observed (data not shown). These data demonstrated that the expressions of most of these immune genes are in line with the microarray results shown in Table 1.

### 3.5 Association of several immune genes with social behavior in C57BL/6 J and DBA/2 J mice

**Figure 4:**
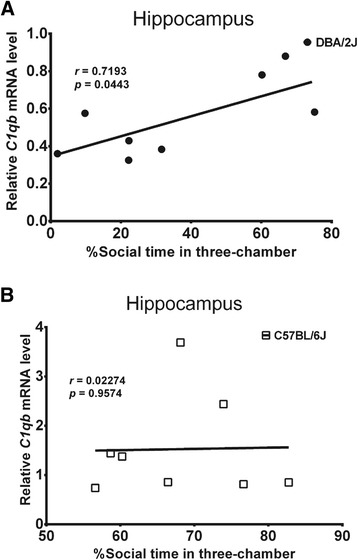
Correlation of *C1qb* expression with social behavior in the C57BL/6 J and DBA/2 J mice. Correlations of the mRNA levels of *C1qb* in the HC with the percentages of social time in the three-chambered test in DBA/2 J (**(A)** *r =* 0.7193, *P <* 0.05) and C57BL/6 J (**(B)** *r =* 0.0227, *P >* 0.05) were analyzed by the Pearson’s method.

We also analyzed the correlations of the mRNA levels of differentially expressed immune genes *C1qb*, *H2-d1*, *Polr3b*, and *Tnfsf13b* with the social behaviors of the C57BL/6 J and DBA/2 J mice in the resident-intruder and three-chambered tests. Interestingly, the mRNA level of *C1qb* in the hippocampus was positively correlated with the percentage of social time in the three-chambered social test in the DBA/2 J mice (*r =* 0.7193, *P <* 0.05; Figure 4A) but not in the C57BL/6 J mice (*r =* 0.0227, *P >* 0.05; Figure 4B). In addition, when the DBA/2 J and C57BL/6 J mice were combined, the mean mRNA level of *H2-d1* in the three brain regions - PFC, hippocampus, and hypothalamus - was positively correlated with the percentage of social time in the three-chambered social test (*r* = 0.5752, *P <* 0.05; data not shown), but no significant correlation was found in the separate strain groups. Furthermore, no significant associations of *Tnfsf13b* and *Polr3b* were observed.

## Discussion

In this study, we combined unbiased bioinformatics with targeted experimental approaches to address what and how immune genes may affect social behaviors in inbred mice, and we discovered several candidate immune genes, such as *H2-d1*, *C1qb, Polr3b*, and *Tnfsf13b*, and the gene networks that they represent. Our data implied the possible involvement of these immune genes in regulating social behavior; the deficit of which is a core symptom of several neuropsychiatric diseases, such as schizophrenia and autism spectrum disorder.

Inbred mouse strains have been extensively used for behavioral research and represent a valid model for studying social behaviors relevant to human neuropsychiatric diseases. We speculated that various immune genes in the brains of different inbred mouse strains may contribute to differences in their behaviors, such as the ability of making social interactions. To test this hypothesis, we started with an analysis of the whole brain Affymetrix microarray data on these strains provided by Phenogen Informatics. Such strategy is based on the notion that gene functions are closely associated with their expression levels. Next, by predicting the association of gene expression with brain morphology and behaviors from the online database, we targeted six differentially expressed immune genes, and we later validated them in the brain regions of the male DBA/2 J and C57BL/6 J mice by RT-qPCR. Out of the six genes, *C1qb*, *H2-d1*, *Polr3b*, and *Tnfsf13b* were validated. In particular, we found that *H2-d1* was significantly decreased in all of the tested brain regions - PFC, hippocampus, and hypothalamus - of DBA/2 J mice, while *C1qb*, *Polr3b* and *Tnfsf13b* may play region-specific roles in the brain.

We further examined the associations of these immune genes with social behaviors among the C57BL/6 J and DBA/2 J mice. Of particular note, the *C1qb* gene was significantly positively associated with the sociability in the DBA/2 J mice, not in the C57BL/6 J mice. This strain-dependent association may be due to the lower expression of *C1qb* in the hippocampus of DBA/2 J than in C57BL/6 J, as the higher threshold of *C1qb* expression in the C57BL/6 J mice may disrupt the linear relationship between gene expression and behavioral output in this strain. Additionally, the average expression of *H2-d1* in the three brain regions was also significantly correlated with sociability in combined strains, but not in separate groups. It should be noted, however, that some of these genes may manifest only a weak effect on the complicate social behavior individually, and therefore, a network of related immune genes may be more relevant to have a joint impact, highlighting the importance of using unbiased approaches to systemically evaluate genetic association with a certain disease feature.

The most significant finding in our study was a cluster of MHCI genes, *H2-d1* in particular, which were differentially expressed in the brains and associated with animal behaviors. Classical MHCI α-chains are encoded by three genes in humans, denoted HLA-A, −B, and -C. In mice, these genes are H2-K, −D, and -L. MHCI molecules are found in an isoform- and region-specific manner throughout the brain [45]. During the past decade, researchers have provided convincing evidence that, as the classical “immune” proteins, MHCI molecules regulate establishment and function of cortical connections, activity-dependent synaptic refinement and pattern formation, long-term depression and motor learning, and homeostatic plasticity by restriction of immune response [24,46,47]. More importantly, the association of MHCI loci with schizophrenia has been confirmed by several cohorts of GWAS data [1,9,46]. These are corroborated by our findings and support the validation of our approach to find out immune-related genetic mechanisms of social deficit in animal models. However, a detailed investigation is warranted to further examine our data.

C1QB is known to mediate the classical complement cascade for synaptic elimination [22,47]. It has been previously demonstrated that retinal C1q expression primes microglia-mediated synaptic pruning during the visual system development [48]. Recently, C1q was found to mediate the elimination of synapses in the hippocampus in multiple sclerosis, leading to memory impairment in patients [49]. Here, we found that *C1qb* was significantly down-regulated in the hippocampus of the DBA/2 J mice, as compared to the C57BL/6 J mice. Additionally, *C1qb* was positively associated with sociability in the DBA/2 J mice. Our data suggests that *C1qb*-mediated synaptic pruning may be less effective in the brains of the DBA/2 J mice, which may further result in the lessened sociability of the DBA/2 J mice.

Another target gene in our study was *Polr3b*, which was significantly up-regulated in the hippocampus of the DBA/2 J mice. Interestingly, mutations of *POLR3A* or *POLR3B* are known to be associated with the neurodegenerative hypomyelinating leukodystrophy spectrum disorders in humans [50-52]. Besides, *POLR3B* was reported to disturb the purine metabolism and contribute to Alzheimer’s disease [53]. The association of the *Polr3b* and its family with social activity of animals has however been unknown so far.

When summarizing the correlated brain structures with various immune genes, we found that one of the most prevalent associations is the length of CC, a key feature of structural abnormality in schizophrenia and autism spectrum disorder. Other inbred mouse strains that show schizophrenia/autism-like endophenotypes, such as 129 and BTBR, are also known to have small or totally absent CC, and have been used to map quantitative trait loci that affect CC size. BTBR strain in particular shows total absence of the CC [54]. Furthermore, the gene disrupted in schizophrenia 1 (*Disc1*) is homozygously inactivated in all 129 mouse substrains, and this genetic mutation may be causally linked to hypo-genesis of CC in these animals [55]. Our predictions that immune genes are associated with brain morphology could provide valuable information to be validated in the future.

In our analysis, the length of CC is negatively associated with *Tnfsf13b*. TNFSF13b (BAFF) is a TNF superfamily member, which acts as a potent B-lymphocyte activator in immune response [56]. Of note, a recent genetic association study, which collected so far the largest cohort of schizophrenic patients and healthy controls, has discovered a significant association of immune genes other than the MHCI loci with schizophrenia and revealed a particular enrichment of genes of B-lymphocyte lineage [1]. Association of B-cell activation-related immune genes with schizophrenia, major depression, and bipolar disorder was also observed in another most recent genetic study [9]. Moreover, a gene involved in B-cell receptor signaling pathway (*PPP3CC*) was suggested to mediate antidepressant response in another pharmacogenetic study [57].

Previous to our work, several immune genes in the brain have been shown to regulate emotional and/or social behaviors of animals. The cytokine interleukin-6 was found to be a critical mediator for the maldevelopment of the fetal brain and the social deficits in the offspring of maternally immune-challenged mice [14], while interleukin-1β was not involved in a similar paradigm [15]. CD38, a transmembrane glycoprotein involved in immune response, has been demonstrated to mediate abnormal oxytocin secretion, maternal nurturing, and social behavior in autism [16]. Another previous study has described a distinct compulsive behavioral phenotype caused by a mutant gene *Hoxb8* that exclusively labels microglia in the brain [21].The results that we provide here help extend the knowledge on the immune-mediated mechanisms of social behavior.

However, our study has several limitations to be considered. Firstly, although we found significant correlations of several immune genes with social behaviors, correlation does not imply causation. Therefore, cautions are called for the interpretation of our data, and more stringent researches to examine the roles of these candidate immune genes are needed. Secondly, some discrepancy between the microarray and RT-qPCR data existed here. For example, we failed to validate the expressions of *Cx3cl1* and *H2-k1*. This may be due to that RT-qPCR is generally more sensitive than microarray but at the same time may cause polymerization bias. And the probes utilized in microarray assay are different from those in RT-qPCR. Moreover, gene expression in the whole brain as detected by microarray may not necessarily parallel with that in the separate brain regions as detected by qPCR in this study. Thirdly, most researches on C57BL/6 and DBA/2 strains focus on male mice, and so did ours. There have been few studies on female mice so far. But gender-specific effect of immune genes on behaviors could exist [58] and is needed to be addressed in the future.

## Conclusions

In summary, we developed a useful approach, by combining bioinformatics with experimental methods and studying inbred mouse strains, to predict novel functions of both known genes and novel genes. Such data provide some hints for further studies to unravel immune-mediated mechanisms of psychiatry-related social deficiency in animals.

## Abbreviations

CC: corpus callosum
MHC: major histocompatibility complex
MPD: mouse phenome database
PFC: prefrontal cortex
